# MAL Overexpression Leads to Disturbed Expression of Genes That Influence Cytoskeletal Organization and Differentiation of Schwann Cells

**DOI:** 10.1177/1759091414548916

**Published:** 2014-09-05

**Authors:** Daniela Schmid, Thomas Zeis, Monia Sobrio, Nicole Schaeren-Wiemers

**Affiliations:** 1 Department of Biomedicine, University Hospital Basel, University of Basel, Switzerland

**Keywords:** myelin and lymphocyte protein (MAL), Schwann cell development, Schwann cell differentiation, peripheral nerve myelination, cytoskeleton

## Abstract

In the developing peripheral nervous system, a coordinated reciprocal signaling between Schwann cells and axons is crucial for accurate myelination. The myelin and lymphocyte protein MAL is a component of lipid rafts that is important for targeting proteins and lipids to distinct domains. MAL overexpression impedes peripheral myelinogenesis, which is evident by a delayed onset of myelination and reduced expression of the myelin protein zero (*Mpz*/*P0*) and the low-affinity neurotrophin receptor *p75^NTR^*. This study shows that MAL overexpression leads to a significant reduction of *Mpz* and *p75^NTR^* expression in primary mouse Schwann cell cultures, which was already evident before differentiation, implicating an effect of MAL in early Schwann cell development. Their transcription was robustly reduced, despite normal expression of essential transcription factors and receptors. Further, the cAMP response element-binding protein (CREB) and phosphoinositide 3-kinase signaling pathways important for Schwann cell differentiation were correctly induced, highlighting that other so far unknown rate limiting factors do exist. We identified novel genes expressed by Schwann cells in a MAL-dependent manner *in vivo* and *in vitro*. A number of those, including S100a4, RhoU and Krt23, are implicated in cytoskeletal organization and plasma membrane dynamics. We showed that S100a4 is predominantly expressed by nonmyelinating Schwann cells, whereas RhoU was localized within myelin membranes, and Krt23 was detected in nonmyelinating as well as in myelinating Schwann cells. Their differential expression during early peripheral nerve development further underlines their possible role in influencing Schwann cell differentiation and myelination.

## Introduction

During development of peripheral nerves, Schwann cells are in tight contact with axons providing mutual trophic support. Accurate Schwann cell development and myelination are controlled and regulated by reciprocal axon-glia interaction (reviewed in Nave & Trapp, 2008). Although axonal signals crucial for development were identified, the process of differentiation and myelination is not yet fully understood. One essential growth factor is neuregulin1 (NRG1), which is shown to be involved in the regulation of the entire Schwann cell lineage, encompassing survival and proliferation of Schwann cell precursors as well as myelination (reviewed in Garratt et al., 2000). Also neurotrophins such as nerve growth factor (NGF), brain-derived neurotrophic factor (BDNF), and neurotrophin 3 (NT3) were shown to be critical for survival, migration, and myelination (reviewed in Xiao et al., 2009). Moreover, the correct localization of proteins and lipids within distinct Schwann cell compartments was shown to influence myelination. Such regulatory mechanisms of signaling and trafficking were often associated with glycolipid- and cholesterol-enriched membrane domains, so-called lipid rafts (Kramer et al., 1999; Schaeren-Wiemers et al., 2004; Schafer et al., 2004).

One important component of lipid rafts is the myelin and lymphocyte protein MAL (VIP17/MVP17), a nonglycosylated integral membrane protein with four transmembrane domains. MAL is localized in polarized epithelial cells of the kidney, stomach, and thyroid gland and was shown to be involved in the sorting and transport of vesicles to the apical membrane (reviewed in Frank, 2000). MAL overexpression leads to pathological apical membrane formation in kidney and stomach, demonstrating that its correct dosage is essential for proper function (Frank et al., 2000). In the nervous system, MAL is expressed by oligodendrocytes in the central nervous system (CNS) and by Schwann cells in the peripheral nervous system (PNS; Schaeren-Wiemers et al., 1995a; Schaeren-Wiemers et al., 1995b). MAL expression in Schwann cells starts already at embryonic day 17, implicating a role in development (Frank et al., 1999). It is expressed by both myelinating and nonmyelinating Schwann cells and is localized not only in compact myelin but also in noncompact regions such as paranodal loops and Schmidt-Lantermann incisures (Erne et al., 2002). Mice overexpressing MAL manifest a retarded maturation of Remak bundles and progressive segregation of unmyelinated axons (Buser et al., 2009b; Frank et al., 2000). Furthermore, mice overexpressing MAL show a delayed onset of myelination, reflected in hypomyelinated fibers during early development (Buser et al., 2009b). Along with changes on the morphological level, altered gene transcription and protein expression were detected in MAL-overexpressing mice. In sciatic nerves of newborn mice, reduced expression of the low-affinity neurotrophin receptor *p75^NTR^* was identified (Buser et al., 2009b). These results suggested that altered *p75^NTR^* expression in MAL-overexpressing mice is the cause of delayed onset of myelination, as distinct expression of *p75^NTR^* has been shown to be important for proper initiation of myelination (Cosgaya et al., 2002). Herein this study, we analyzed particular signaling pathways known to be relevant for Schwann cell differentiation by investigating primary mouse Schwann cell cultures treated with either forskolin or NRG1 (Schmid et al., 2014). A whole genome expression profiling was further performed to identify MAL-dependent differentially expressed transcripts.

## Material and Methods

### Mouse Line

The MAL-overexpressing mouse line was generated by introducing a 34-kb insert of the cosmid pTCF-MAL2.1, containing the *Mal* gene, which is flanked by 8 kb of upstream nontranscribed region (Frank et al., 2000; Magyar et al., 1997). MAL is overexpressed in a tissue- and cell-specific manner, and pathological alterations were previously described (Buser et al., 2009b; Frank et al., 2000). MAL-overexpressing mice were routinely bred with C57/Bl6 mice, and heterozygous mice with respective wild-type littermates were used in this study. All mice were kept under standard specific pathogen-free conditions, housed, and treated according to the guidelines for care and use of experimental animals of the veterinary office of the Canton of Basel-Stadt.

### Primary Mouse Schwann Cell Cultures

Schwann cells were prepared as described earlier (Schmid et al., 2014). Sciatic nerves from postnatal day 1 (P1) mice were dissociated with 0.4% collagenase and 0.125% trypsin, Dulbecco’s Modified Eagle Medium (DMEM; D6546; Sigma-Aldrich) supplemented with 10% fetal bovine serum (FBS) was added, and cells were seeded onto 24-well plates (Primaria™, BD Bioscience). A day after, Schwann cells were treated with 10 µM cytosine β-d-arabinofuranoside (AraC) twice for 24 h to reduce fibroblast proliferation. Schwann cells were passaged, and cells of the respective genotype were pooled and cultured in DMEM containing 10% FBS, unless not otherwise stated. For mRNA expression analysis, primary Schwann cells were seeded at a density of 25,000 cells/well. For immunofluorescence analysis, 15,000 Schwann cells were seeded on poly-d-lysine and laminin-coated glass coverslips in a 40-µl drop. Purity of mouse Schwann cell cultures determined by immunofluorescent stainings for p75^NTR^ and S100β revealed more than 85% enrichment (information about antibodies in Supplementary Table 1).

For Schwann cell differentiation assay, cells were stimulated with 20 µM forskolin (Sigma-Aldrich) in DMEM supplemented with 10% FBS for 24 h as described earlier (Schmid et al., 2014). For investigation of the phosphoinositide 3-kinase (PI3-kinase) activity, Schwann cells were cultured in DMEM supplemented with 1% FBS for 15 h and then treated with 2.5 nM human recombinant heregulin-β1 (herein called neuregulin1; Sigma-Aldrich) in DMEM supplemented with 1% FBS for 15 min at 37°C (Ogata et al., 2004).

### Expression Analysis

**Figure 1.**
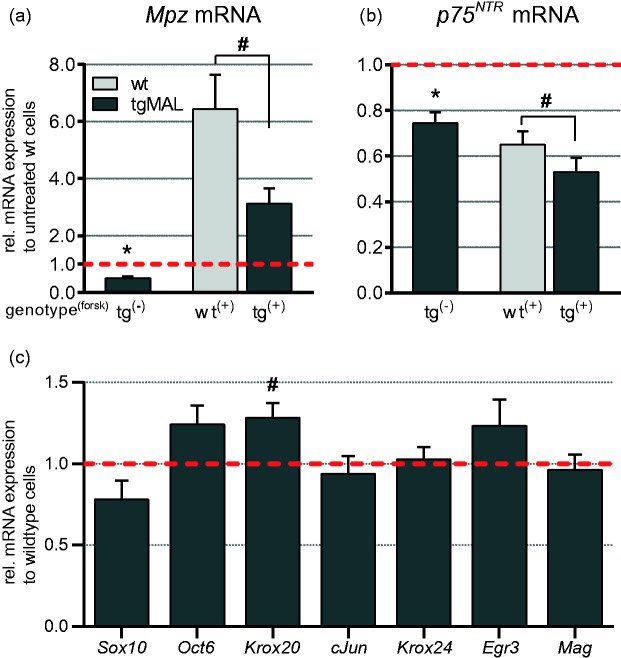
Differential expression analysis in primary Schwann cell cultures of MAL-overexpressing and wild-type mice. (a, b) Schwann cells derived from P1 mice were cultured in the presence or absence of 20 µM forskolin for 24 h and analyzed by qRT-PCR. (a) A substantial induction of *Mpz* expression was investigated for wild-type and MAL-overexpressing cells upon treatment; however, the expression of *Mpz* was significantly reduced in MAL-overexpressing Schwann cells compared with wild-type cells. (b) Under both conditions, *p75^NTR^* mRNA level was reduced in MAL-overexpressing mice. Upon treatment, *p75^NTR^* expression was decreased in both genotypes. Data were normalized to the expression of *60s*, and values for unstimulated wild-type samples were set to 1. Data represent the mean of at least 20 independent experiments for unstimulated condition and at least 9 independent experiments for stimulated condition. The error bars indicate the *SEM*. (c) Gene transcripts implicated in Schwann cell development and differentiation were investigated in untreated Schwann cells by qRT-PCR. Data were normalized to the expression of *60s*, and wild-type values were set to 1. The columns show the mean value of at least nine independent experiments, and the error bars indicate the *SEM*. *p < 0.0001. #*p* < 0.01.

Schwann cells were washed with phosphate-buffered saline (PBS), and total RNA was isolated using RNeasy Micro Kit (Qiagen) according to the manufacturer’s protocol. First-strand cDNA synthesis was performed using Transcriptor Reverse Transcriptase (Roche) and random hexamer primers (Roche). Primers for quantitative reverse transcriptase-polymerase chain reaction (qRT-PCR) were designed with Clone Manager software (Science and Educational Software) or with NCBI PrimerBLAST. Primer pairs were chosen to overlap exon/intron junctions to prevent amplification of genomic DNA (Supplementary Table 2). qRT-PCR was performed on the 7500 Fast Real-time PCR System (Applied Biosystems) with Fast SYBR Master Mix (Applied Biosystems). The acquired mRNA copy numbers were normalized to the one of the 60S ribosomal protein subunit L13a. For the graph of *Mpz* and *p75^NTR^*, values represent the mean of at least 20 independent experiments for unstimulated and at least 9 independent experiments for stimulated primary Schwann cells [Figure 1(a) and (b)]. For qRT-PCR analysis of *Sox10*, *Oct6*, *Krox20*, *cJun*, *Krox24*, *Egr3*, and *Mag*, data represent the mean of at least nine independent experiments [Figure 1(c)]. For the *in vivo* analysis, sciatic nerves of two MAL-overexpressing mice and respective wild-type littermates were pooled, and total RNA was isolated with the ZR RNA MicroPrep™ Kit (Zymo Research). First-strand cDNA synthesis was performed using GoScript™ reverse transcriptase (Promega) and random hexamer primers (Roche). qRT-PCR was performed on the ViiA™ 7 Real-time PCR System (Applied Biosystems) with KAPA Sybr Fast Master Mix (Kapa Biosystems). The acquired mRNA copy numbers were normalized to the one of the 60S ribosomal protein subunit L13a.

### Determination of PI3-Kinase Activity by Measuring Phospho-Akt Levels

To determine the phospho-Akt levels *in vivo*, sciatic nerves of four newborn MAL-overexpressing and wild-type littermates were collected per experiment. Homogenization of the nerve tissues was performed in lysis buffer (20 mM phosphate buffer pH 7.4, 250 mM NaCl, 1% Triton X-100, and 0.1% sodium dodecyl sulfate [SDS]) with complete protease inhibitor mixture (Roche), 5 mM NaF, and 1 mM Na_3_VO_4_ using Lysing Matrix D tubes (MP Biomedicals) in a FastPrep FP120 homogenizer (MP Biomedicals) for 30 s at 6.0 m/s. The homogenate was incubated in 4× Laemmle buffer and 20% β-mercaptoethanol for 1 h at 37°C. Protein separation on a 10% SDS-polyacrylamide gel electrophoresis (PAGE) gel, protein transfer, and antibody incubations were performed as described earlier (Buser et al., 2009b). Immunofluorescent signal detection was investigated by the Odyssey™ Imaging System (LI-COR) and quantified using ImageJ. The expression levels were normalized to the one of β-actin.

### Whole Genome Expression Profiling

MAL-overexpressing and wild-type Schwann cells were cultured and stimulated with 20 µM forskolin for 24 h (Schmid et al., 2014). Nine experimental samples were investigated per condition, derived from five independent experiments. Total RNA was isolated using RNeasy Micro Kit (Qiagen) according to the manufacturer’s protocol. All RNA samples had an RNA integrity number (RIN) of above 8, verified with the Agilent Bioanalyzer system (Agilent Technologies). RNA amplification, biotinylation, *in vitro* transcription, and cRNA hybridization were performed as described earlier (Kinter et al., 2013; Schmid et al., 2014). MouseWG-6 v2.0 Expression BeadChips from Illumina were scanned using the iScan Reader (Illumina), and global median normalization of gene expression was performed with the GenomeStudio software (version 2011.1, Illumina). All data passed the quality control analysis as assessed by the Illumina on-board software (GenomeStudio, version 2011.1) and principal component analysis (PCA; Partek Genomics Suite, version 6.6, Partek Inc.). Significantly differentially expressed genes were further analyzed with the Ingenuity Pathway Analysis (IPA) software (Ingenuity Systems) and the Database for Annotation, Visualization and Integrated Discovery (DAVID, version 6.7, DAVID Bioinformatics Resources; Huang da, Sherman, & Lempicki, 2009).

### Immunofluorescent Microscopy

Cultured Schwann cells were rinsed with PBS and fixed in 4% paraformaldehyde in PBS for 15 min. After washing three times with PBS for 30 min, unspecific binding sites were impeded by incubation with blocking buffer containing 1% normal donkey serum (Chemicon Int.), 2% cold fish skin gelatin (Sigma-Aldrich), and 0.15% Triton X-100 (Sigma-Aldrich) in PBS for 1 h at room temperature. Primary antibodies (Supplementary Table 1) were incubated in blocking buffer at 4°C overnight. Fluorochrome-conjugated secondary antibodies were diluted in blocking buffer and incubated for 1 h at room temperature. Stained sections were embedded in FluorSave (Calbiochem). For *in vivo* immunofluorescent stainings, 10-µm tissue sections of fresh-frozen sciatic nerves of 3-month-old MAL-overexpressing mice and wild-type littermates were mounted on Superfrost Plus slides (Thermo Scientific), dried at room temperature, and fixed in 4% paraformaldehyde for 15 min. After washing three times for 30 min with PBS, slides were incubated in 80% ethanol for 1 h and thereafter washed with PBS. Further procedure was performed as described earlier. Immunofluorescent images were acquired with either a Nikon A1R microscope (40× objective, numerical aperture 1.30) or with a Zeiss LSM710 microscope (40× objective, numerical aperture 1.30; 63× objective, numerical aperture 1.40). For quantification of phospho-Akt staining, 400 nonoverlapping images per genotype and condition were acquired. For each image, a threshold level of 2 to 255 was set, and the average staining intensity was recorded. The mean of this value was determined for each coverslip (50 images) and then for each condition. The average staining intensity was calculated over three independent experiments. Image processing was performed with ImageJ 1.47 b software and Adobe Photoshop software (version CS5.1).

### Statistical Analysis

For qRT-PCR expression analysis *in vitro*, values represent the mean of itemized independent experiments, and error bars indicate the *SEM*. Statistical analysis was performed using a Student’s *t* test for paired groups. For qRT-PCR expression analysis *in vivo*, values represent the mean of at least four experimental samples, and the error bars indicate the *SD*. Statistical analysis was performed using a Student’s *t* test. Quantification of immunofluorescent stainings of phospho-Akt is based on three independent experiments. Data were analyzed by a two-way analysis of variance (ANOVA), and error bars indicate the *SEM*. For Western blot analysis, data are shown as mean of three independent experiments, and error bars indicate the *SD*. Statistical quantification was performed by a Student’s *t* test for paired groups. Microarray data analysis was performed using Partek Genomic Suite software. Differentially expressed transcripts were identified by performing a three-way ANOVA (Genotype, Treatment, and Array), and *p* values were adjusted using the false discovery rate (FDR) method to correct for multiple comparisons (Benjamini & Hochberg, 1995). Data are shown as mean, and error bars indicate the *SD*.

## Results

### MAL Overexpression Leads to Decreased *Mpz* and *p75^NTR^* Expression in Primary Schwann Cell Cultures

From our developmental study on PNS myelination, we observed that MAL overexpression retarded the onset of myelination (Buser et al., 2009b). Therefore, we investigated whether Schwann cells overexpressing MAL are less responsive to stimuli for Schwann cell differentiation *in vitro*. Schwann cells isolated from sciatic nerves of P1 MAL-overexpressing mice and wild-type littermates were treated for 24 h with 20 µM forskolin, a generally used reagent to activate the cAMP response element-binding protein (CREB) and to induce the expression of myelin-related genes (Lemke & Chao, 1988; Monje et al., 2009; Parkinson et al., 2003; Schmid et al., 2014). Subsequently, differential gene expression was analyzed by qRT-PCR. Schwann cells derived from MAL-overexpressing mice showed a robust overexpression of *Mal* mRNA also *in vitro* under both treated and untreated conditions (data not shown). Schwann cell differentiation upon forskolin treatment was determined by the induction of myelin protein zero (*Mpz*/*P0*) expression. In wild-type Schwann cells, a 6.5-fold induction of *Mpz* mRNA expression was detected upon stimulation with forskolin [Figure 1(a)]. The increase of *Mpz* expression upon treatment was comparable between MAL-overexpressing and wild-type cells. However, Schwann cells derived from MAL-overexpressing mice showed an overall 50% reduction of *Mpz* mRNA expression under both unstimulated (*p* < 0.0001) and stimulated (*p* < 0.007) conditions [Figure 1(a)].

Expression of *p75^NTR^* in wild-type Schwann cells was reduced approximately 35% by forskolin stimulation [Figure 1(b), wt^+^], corresponding to the *in vivo* situation in which *p75^NTR^* expression was downregulated during myelination (Buser et al., 2009b). Along with the reduced expression of *Mpz*, untreated Schwann cells overexpressing MAL manifested a 25% reduction in *p75^NTR^* mRNA expression compared with wild-type cells [*p* < 0.0001; Figure 1(b)], confirming the *in vivo* situation (Buser et al., 2009b). As for wild-type cells, forskolin treatment led to a 30% decrease of *p75^NTR^* expression level in MAL-overexpressing Schwann cells [Figure 1(b)], and still, a reduction of 20% compared with wild-type cells could be detected (*p* < 0.006).

The expression levels of *Sox10*, *Oct6*, *Krox20*, *cJun*, *Krox24*, *Egr3*, and *Mag* were investigated in primary unstimulated mouse Schwann cell cultures [Figure 1(c)] to determine whether the reduced mRNA level of *Mpz* and *p75^NTR^* was possibly caused by the altered expression of transcription factors known to modulate their expression (Bermingham et al., 1996; Gao et al., 2007; Jaegle et al., 1996; Peirano et al., 2000; Topilko et al., 1994). In MAL-overexpressing Schwann cells, *Sox10* expression was slightly reduced (*p* < 0.08), validating previous *in vivo* observations (Buser et al., 2009b). However, expression of the transiently activated transcription factor *Oct6* was increased in MAL-overexpressing Schwann cells (*p* < 0.06). Furthermore, the expression of *Krox20* was significantly increased in MAL-overexpressing Schwann cells compared with wild-type cells (*p* < 0.005). As a negative regulator for myelination, the expression of *cJun* was investigated (Parkinson et al., 2008), although no altered transcription was detected in MAL-overexpressing Schwann cells. The transcription factors *Krox24* and *Egr3* were investigated due to their ability to modulate *p75^NTR^* expression (Gao et al., 2007), but the expression levels of both transcripts were not significantly changed. In contrast to *Mpz*, MAL overexpression did not influence the expression of the myelin-associated glycoprotein (*Mag*), which is also early expressed during Schwann cell development.

Our results demonstrate that the activation of the CREB signaling pathway is not affected by MAL overexpression and did not improve reduced *Mpz* and *p75^NTR^* expression in MAL-overexpressing Schwann cells. Our analysis further revealed that *Mpz* and *p75^NTR^* were already reduced in unstimulated Schwann cells overexpressing MAL, suggesting that alteration in gene regulation due to MAL overexpression is taking place at an early stage of Schwann cell development.

### Phosphorylation of Akt Is Not Altered in MAL-Overexpressing Schwann Cells

**Figure 2.**
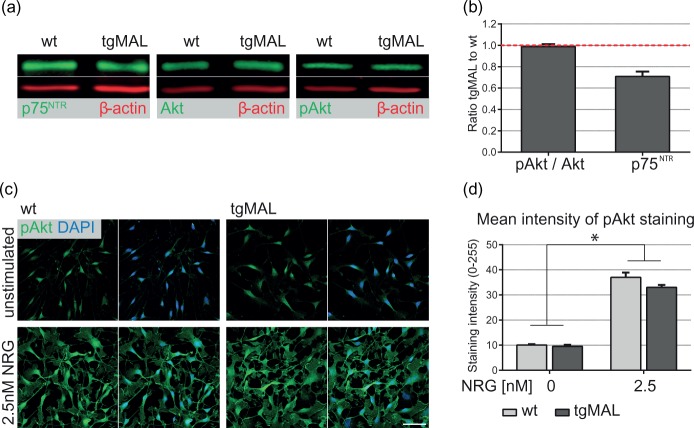
Investigation of Akt phosphorylation in sciatic nerves and in primary mouse Schwann cell cultures. (a) Homogenates of sciatic nerves derived from newborn MAL-overexpressing mice and wild-type littermates were analyzed by quantitative Western blot analysis. A representative blot for p75^NTR^, Akt, and phosphorylated Akt (Ser 473) was used for illustration. (b) The activation of Akt pathway, indicated by the ratio phospho-Akt to Akt showed no alteration in MAL-overexpressing nerves, whereas p75^NTR^ protein expression was reduced (*p* = 0.08). All values were normalized to the expression of β-actin and are shown as a ratio to the respective wild-type expression. Columns represent the mean value of three independent experiments, and the error bars indicate the *SD*. (c) Immunofluorescence stainings on primary MAL-overexpressing and wild-type Schwann cells were performed with an antibody recognizing phosphorylated Akt (Ser 473). Cells were stimulated with 2.5 nM neuregulin1 for 15 min, and an induction of Akt phosphorylation was detected for both genotypes. DAPI as a nuclear marker was used for counterstaining. Bar: 50 µm. (d) Quantification of the mean signal intensity of phospho-Akt in unstimulated and stimulated Schwann cells revealed a significant 3.5-fold induction upon neuregulin1 treatment. Under both conditions, phosphorylation of Akt was not altered in MAL-overexpressing mice. A total of 400 pictures were analyzed per condition and genotype. Data represent the mean of three independent experiments, and error bars indicate the *SEM*. *p < 0.0001; NRG: neuregulin1.

As the activation of the CREB signaling pathway seemed to be unaffected by MAL overexpression, we investigated whether the decreased *Mpz* expression in MAL-overexpressing mice is caused by a reduced activation of the PI3-kinase pathway, a major signaling pathway in Schwann cells required for myelination. Protein expression levels of total Akt and phosphorylated Akt, a key effector of PI3-kinase, were measured in sciatic nerves of newborn mice by quantitative Western blot analysis. In MAL-overexpressing mice, both total Akt and phosphorylated Akt were expressed at normal levels [Figure 2(a)]. To determine the activation of Akt, the ratio of phosphorylated Akt to total Akt was assessed [Figure 2(b)]. This ratio was similar between MAL-overexpressing and wild-type nerves, indicating that delayed onset of myelination in MAL-overexpressing mice was not due to reduced Akt phosphorylation. Investigation of p75^NTR^ expression was included as an internal control, and its expression was decreased by 30% in newborn MAL-overexpressing mice, validating previous observations (Buser et al., 2009b).

In parallel, we investigated Akt phosphorylation in primary mouse Schwann cell cultures. MAL-overexpressing and wild-type Schwann cells were treated with 2.5 nM NRG1 for 15 min to induce Akt phosphorylation *in vitro*, and the activation of Akt was determined by immunofluorescence for phosphorylated Akt [Figure 2(c) and (d)]. We analyzed the mean intensity of the phospho-Akt immunofluorescence in stimulated and unstimulated cells of both genotypes. NRG1 treatment resulted in a significant 3.5-fold induction of phosphorylated Akt in wild-type Schwann cells [*p* < 0.0001; Figure 2(c) and (d)], which is consistent with previous results in rat Schwann cells (Ogata et al., 2004). A comparable induction was observed for Schwann cells derived from MAL-overexpressing mice. In MAL-overexpressing Schwann cells, phosphorylation of Akt was unaltered in either condition compared with wild-type cells, which is in line with the *in vivo* observation. Schwann cell morphology was not changed during NRG1 treatment, verified by a comparable distribution of p75^NTR^ staining (data not shown). From our data, we conclude that delayed onset of myelination in MAL-overexpressing mice was not caused by impaired activation of the PI3-kinase/Akt pathway, as phosphorylation of Akt was unaltered in sciatic nerves of newborn mice overexpressing MAL. Further, comparable staining intensities of phosphorylated Akt were detected between MAL-overexpressing and wild-type Schwann cell cultures, indicating that induction of the PI3-kinase/Akt pathway is functional.

### Differential Gene Expression Analysis of Genes Implicated in Schwann Cell Development in MAL-Overexpressing Schwann Cells

To determine whether the transcriptional regulation of additional genes influencing the onset of myelination was altered due to MAL overexpression, a whole genome expression assay covering more than 45,000 transcripts was performed for primary mouse Schwann cell cultures. In total, nine independent samples of untreated and forskolin-treated Schwann cells derived from MAL-overexpressing mice and wild-type littermates were analyzed. One coding DNA sequence may be represented by several distinct oligonucleotides (probes). For all examinations, probe-specific analysis was performed, allowing to identify MAL-dependent differentially expressed transcripts with high confidence. To investigate the effect of MAL overexpression on global gene expressions, data were visualized by an initial PCA generated in Partek Genomics Suite (Supplementary Figure 1). This analysis revealed that treatment with forskolin was the major source of variability. Based on the genotype, no distinct clustering of MAL-overexpressing and wild-type samples was detected, suggesting that only a small number of genes were differentially regulated in Schwann cells overexpressing MAL.

In a first step, we analyzed the mRNA expression levels of transcription factors that are implicated in Schwann cell development, differentiation, and myelination (Table 1, A). This analysis revealed a small but significant reduction of *Sox10* mRNA expression in MAL-overexpressing Schwann cells. In general, however, transcription factors known to positively or negatively modulate Schwann cell differentiation and myelination were not altered in MAL-overexpressing Schwann cells. Analysis of the expression pattern of receptors implicated in neuregulin or neurotrophin signaling (e.g., *ErbB2*, *ErbB3*, *TrkB*, *TrkC*) also revealed no significant changes in MAL-overexpressing Schwann cells (Table 1, B). However, *p75^NTR^* mRNA levels were significantly reduced, validating data obtained by qRT-PCR.

**Table 1.** Genes Implicated in Schwann Cell Development and Differentiation in MAL-Overexpressing and Wild-type Schwann Cell Cultures.

Common name	Entrez ID	tgMAL:wt	*p*
**A** Transcription factor			
Early Growth Response 1 (Krox24)	*Egr1*	0.933	n.s.
Early Growth Response 2 (Krox20)	*Egr2*	0.894	n.s.
Early Growth Response 3	*Egr3*	1.045	n.s.
Early Growth Response 3	*Egr3*	0.915	n.s.
Inhibitor of DNA binding 2	*Id2*	0.888	n.s.
Inhibitor of DNA binding 2	*Id2*	0.981	n.s.
Inhibitor of DNA binding 4	*Id4*	1.135	n.s.
Jun Oncogen (cJun)	*Jun*	0.928	n.s.
Nab1, EGR-1-binding protein 1	*Nab1*	0.961	n.s.
Nab1, EGR-1-binding protein 1	*Nab1*	1.026	n.s.
Nab1, EGR-1-binding protein 1	*Nab1*	1.006	n.s.
Nab1, EGR-1-binding protein 1	*Nab1*	1.004	n.s.
Nab2, EGR-1-binding protein 2	*Nab2*	1.067	n.s.
Nuclear factor of activated T cells, cytoplasmic 3 (Nfat4)	*Nfatc3*	0.971	n.s.
Nuclear factor of activated T cells, cytoplasmic 3 (Nfat4)	*Nfatc3*	1.072	n.s.
Nuclear factor of activated T cells, cytoplasmic 3 (Nfat4)	*Nfatc3*	1.052	n.s.
Nuclear factor of activated T cells, cytoplasmic 3 (Nfat4)	*Nfatc3*	0.976	n.s.
Nuclear factor of activated T cells, cytoplasmic 4 (Nfat3)	*Nfatc4*	1.002	n.s.
Paired Box Gene 3	*Pax3*	not detected	
POU Domain, class 3, Transcription factor 1 (Oct6, SCIP)	*Pou3f1*	0.873	n.s.
**SRY-box Containing Gene 10**	***Sox10***	**0.885**	**0.0103**
**SRY-box Containing Gene 2**	***Sox2***	**0.848**	**0.0039**
SRY-box Containing Gene 2	*Sox2*	1.000	n.s.
Yin Yang 1	*Yy1*	not detected	
**B** Receptor			
A Disintegrin And Metallopeptidase Domain 22	*Adam22*	1.065	n.s.
v-Erb-b2 Erythroblastic Leukemia Viral Oncogene Homolog 2	*Erbb2*	0.937	n.s.
v-Erb-b2 Erythroblastic Leukemia Viral Oncogene Homolog 3	*Erbb3*	0.976	n.s.
G protein-coupled receptor 126	*Gpr126*	0.982	n.s.
**Nerve Growth Factor Receptor (p75^NTR^)**	***Ngfr***	**0.896**	**0.0016**
**Nerve Growth Factor Receptor (p75^NTR^)**	***Ngfr***	**0.886**	**0.0053**
Nerve Growth Factor Receptor (p75^NTR^)	*Ngfr*	0.930	n.s.
Neurotrophic Tyrosine Kinase, Receptor, Type 2 (TrkB)	*Ntrk2*	1.069	n.s.
Neurotrophic Tyrosine Kinase, Receptor, Type 3 (TrkC)	*Ntrk3*	1.008	n.s.
Neurotrophic Tyrosine Kinase, Receptor, Type 3 (TrkC)	*Ntrk3*	1.064	n.s.
**C** Myelin membrane			
**2',3'-cyclic nucleotide 3' phosphodiesterase (CNPase)**	***Cnp***	**0.882**	**0.0037**
**2',3'-cyclic nucleotide 3' phosphodiesterase (CNPase)**	***Cnp***	**0.869**	**0.0140**
Gap Junction Protein α1 (Connexin 43)	*Gja1*	0.986	n.s.
Gap Junction Protein α4 (Connexin 37)	*Gja4*	1.129	n.s.
Gap Junction Protein β1 (Connexin 32)	*Gjb1*	not detected	
Gap Junction Protein β2 (Connexin 26)	*Gjb2*	1.024	n.s.
Gap Junction Protein β2 (Connexin 26)	*Gjb2*	1.127	n.s.
Gap Junction Protein γ3 (Connexin 29)	*Gjc3*	not on the array	
Lipin 1	*Lpin1*	1.031	n.s.
Lipin 1	*Lpin1*	1.001	n.s.
Myelin-Associated Glycoprotein	*Mag*	0.930	n.s.
**Myelin and Lymphocyte**	***Mal***	**21.533**	**<0.0001**
**Myelin and Lymphocyte**	***Mal***	**12.687**	**<0.0001**
**Myelin and Lymphocyte**	***Mal***	**1.168**	**<0.0001**
Myelin Basic Protein	*Mbp*	0.960	n.s.
Myelin Basic Protein	*Mbp*	0.913	n.s.
Myelin Basic Protein	*Mbp*	0.974	n.s.
Myelin Basic Protein	*Mbp*	0.992	n.s.
Myelin Basic Protein	*Mbp*	0.974	n.s.
Myelin Protein Zero (P0)	*Mpz*	0.961	n.s.
**Myelin Protein Zero (P0)**	***Mpz***	**0.669**	**0.0003**
Neurofascin	*Nfasc*	1.025	n.s.
**Plasmolipin**	***Pllp***	**0.801**	**0.0023**
Peripheral Myelin Protein 2	*Pmp2*	1.120	n.s.
Peripheral Myelin Protein 22	*Pmp22*	1.147	n.s.
Peripheral Myelin Protein 22	*Pmp22*	1.169	n.s.
Periaxin	*Prx*	0.987	n.s.
Periaxin	*Prx*	1.008	n.s.
**D** Cytoplasma			
Growth Associated Protein 43	*Gap43*	1.033	n.s.
Glial Fibrillary Acidic Protein	*Gfap*	0.870	n.s.
Glial Fibrillary Acidic Protein	*Gfap*	0.912	n.s.
S100 β	*S100b*	0.983	n.s.
**E** Extra Cellular Matrix (ECM) /Polarization			
Agrin	*Agrn*	0.991	n.s.
Cell Division cycle 42	*Cdc42*	0.994	n.s.
Cadherin2 (Ncad)	*Cdh2*	0.996	n.s.
**Collagen typeII, α1**	***Col2a1***	**1.454**	**0.0014**
**Collagen typeII, α1**	***Col2a1***	**1.419**	**0.0027**
**Collagen typeII, α1**	***Col2a1***	**1.330**	**0.0003**
Collagen typeIV, α1	*Col4a1*	1.116	n.s.
**Collagen typeIV, α5**	***Col4a5***	**1.154**	**0.0070**
Collagen typeV, α1	*Col5a1*	0.999	n.s.
Collagen typeV, α2	*Col5a2*	1.075	n.s.
Collagen typeV, α2	*Col5a2*	0.917	n.s.
Collagen typeV, α2	*Col5a2*	1.116	n.s.
Collagen typeV, α3	*Col5a3*	1.005	n.s.
Collagen typeVI, α1	*Col6a1*	1.093	n.s.
Collagen typeVI, α1	*Col6a1*	1.015	n.s.
Collagen typeVI, α1	*Col6a1*	1.100	n.s.
Collagen typeVI, α2	*Col6a2*	1.102	n.s.
Collagen typeVI, α2	*Col6a2*	1.037	n.s.
Collagen typeVI, α3	*Col6a3*	1.014	n.s.
Collagen typeVI, α3	*Col6a3*	1.082	n.s.
**Dystroglycan**	***Dag1***	**0.838**	**0.0001**
**Dystroglycan**	***Dag1***	**0.891**	**0.0113**
Disks Large Homolog 1	*Dlg1*	1.005	n.s.
Dedicator of Cytokinesis Protein 7	*Dock7*	1.000	n.s.
Dedicator of Cytokinesis Protein 7	*Dock7*	0.963	n.s.
Dystrophin-related Protein 2	*Drp2*	1.044	n.s.
**Dystrobrevin α**	***Dtna***	**0.799**	**<0.0001**
**Dystrobrevin α**	***Dtna***	**0.843**	**0.0100**
**Dystrobrevin α**	***Dtna***	**0.885**	**0.0186**
**Dystrobrevin α**	***Dtna***	**0.851**	**0.0003**
Dystrobrevin α	*Dtna*	0.953	n.s.
Dystrobrevin β	*Dtnb*	1.014	n.s.
Gliomedin	*Gldn*	0.936	n.s.
Histone Deacetylase	*Hdac1*	not on the array	
Histone Deacetylase	*Hdac2*	1.031	n.s.
Histone Deacetylase	*Hdac2*	0.981	n.s.
Perlecan (Heparan Sulfate Proteoglycan 2)	*Hspg2*	1.043	n.s.
Perlecan (Heparan Sulfate Proteoglycan 2)	*Hspg2*	1.010	n.s.
Perlecan (Heparan Sulfate Proteoglycan 2)	*Hspg2*	0.978	n.s.
Integrin α1	*Itga1*	1.073	n.s.
Integrin α1	*Itga1*	1.107	n.s.
Integrin α6	*Itga6*	0.977	n.s.
Integrin α6	*Itga6*	0.964	n.s.
Integrin α7	*Itga7*	0.861	n.s.
Integrin β1	*Itgb1*	1.009	n.s.
Integrin β1	*Itgb1*	1.077	n.s.
Integrin β4	*Itgb4*	0.899	n.s.
Integrin β4	*Itgb4*	0.894	n.s.
Laminin α1	*Lama1*	1.029	n.s.
Laminin α2	*Lama2*	0.994	n.s.
Laminin α4	*Lama4*	1.001	n.s.
Laminin α5	*Lama5*	1.068	n.s.
Laminin β1	*Lamb1-1*	0.937	n.s.
Laminin β2	*Lamb2*	1.020	n.s.
Laminin γ1	*Lamc1*	1.060	n.s.
Laminin γ1	*Lamc1*	1.038	n.s.
Laminin γ2	*Lamc2*	1.045	n.s.
Laminin γ2	*Lamc2*	1.000	n.s.
Laminin γ2	*Lamc2*	0.983	n.s.
Membrane Protein, Palmitoylated 5 (Pals1)	*Mpp5*	0.935	n.s.
Neural Cell Adhesion Molecule (CD56)	*Ncam1*	1.059	n.s.
Neural Cell Adhesion Molecule (CD56)	*Ncam1*	1.081	n.s.
Neural Cell Adhesion Molecule (CD56)	*Ncam1*	1.102	n.s.
Nidogen 1 (Entactin)	*Nid1*	0.907	n.s.
Nidogen 1 (Entactin)	*Nid1*	1.003	n.s.
Nidogen 2	*Nid2*	0.934	n.s.
Partitioning Defective 3 Homolog (Par3)	*Pard3*	1.002	n.s.
Partitioning Defective 3 Homolog (Par3)	*Pard3*	0.970	n.s.
Partitioning Defective 3 Homolog (Par3)	*Pard3*	1.071	n.s.
Phosphatase and Tensin Homolog	*Pten*	0.991	n.s.
Phosphatase and Tensin Homolog	*Pten*	0.996	n.s.
Ras-Related C3 Botulinum Substrate 1	*Rac1*	1.045	n.s.
Ras Homolog Family Member A	*Rhoa*	not detected	
Ras Homolog Family Member B	*Rhob*	0.975	n.s.
Sarcoglycan α	*Sgca*	not detected	
Sarcoglycan δ	*Sgcd*	0.989	n.s.
Sarcoglycan ɛ	*Sgce*	not detected	
Sarcoglycan γ	*Sgcg*	not detected	
Structural Maintenance of Chromosomes 3 (Bamacan)	*Smc3*	1.010	n.s.
Syntrophin acidic 1	*Snta1*	0.982	n.s.
Syntrophin acidic 1	*Snta1*	1.011	n.s.
Syntrophin basic 1	*Sntb1*	0.990	n.s.
Syntrophin basic 2	*Sntb2*	0.907	n.s.
Syntrophin basic 2	*Sntb2*	0.923	n.s.
Syntrophin basic 2	*Sntb2*	1.029	n.s.
Syntrophin γ1	*Sntg1*	not detected	
Syntrophin γ2	*Sntg2*	not detected	
Utrophin	*Utrn*	not detected	
**F** Varia			
Leucine-Rich Repeat LGI family, Member 4	*Lgi4*	0.901	n.s.
Leucine-Rich Repeat LGI family, Member 4	*Lgi4*	0.930	n.s.

*Note.* A three-way analysis of variance of microarray data revealed that the mRNA expression of most of the investigated genes implicated in Schwann cell development, differentiation, and myelination were unaltered in MAL-overexpressing cells. Based on a probe-specific analysis, several probes per coding DNA sequence were analyzed separately. The expression was normalized to the global median, and data indicate the ratio of expression in MAL-overexpressing to wild-type Schwann cells. Unadjusted *p* ≤ 0.01 was accounted as significant (bold); n.s. = not significant.

Analysis of myelin-related gene transcripts showed the expected increase of *Mal* in MAL-overexpressing Schwann cells, and the reduced expression of *Mpz* upon MAL overexpression could be confirmed (Table 1, C). In addition, a significant reduction of 2',3'-cyclic nucleotide 3' phosphodiesterase (*Cnp*) and the proteolipid protein plasmolipin (*Pllp*) was identified in MAL-overexpressing Schwann cells, which was validated by qRT-PCR (Supplementary Figure 2). Like MAL, plasmolipin is a component of lipid rafts and shares some structural similarities (Bosse et al., 2003; Magyar et al., 1997). Both tetraspans are expressed in the CNS, the PNS and in the apical membrane of tubular epithelial cells in the kidney (Cochary et al., 1990; Zacchetti et al., 1995), underlining a putative functional relationship between MAL and plasmolipin. The expression of other myelin-related transcripts seemed not to be affected in MAL-overexpressing Schwann cells.

A closer look at gene sequences associated with the extracellular matrix (ECM) revealed significant reduction of α-dystrobrevin (*Dtna*) and dystroglycan (*Dag1*), two members of the dystrophin-glycoprotein complex (Table 1, E). Transcripts of other members of this complex were either expressed below background levels or were unaltered (*Dtnb*, *Sgcd*, *Snta1-Sntg2*, and *Utrn*). This was also the case for the different isoforms of integrin and laminin as well as for entactin (nidogen, *Nid1/2*) and the proteoglycans agrin (*Agrn*), perlecan (*Hspg2*), and bamacan (*Smc3*). However, significantly increased expression levels of collagen type II α1 (*Col2a1*) and collagen type IV α5 (*Col4a5*) transcripts were detected in MAL-overexpressing Schwann cells, whereas comparable expression levels for collagen type V and VI were observed. Investigation of genes implicated in polarity revealed normal mRNA expression levels in MAL-overexpressing Schwann cells (*Dlg1*, *Pard3*, and *Dock7*).

In summary, we identified that transcripts of a small number of genes known to be involved in Schwann cell development were differentially expressed in MAL-overexpressing Schwann cells, but the consistent reduction of *Mpz* and *p75^NTR^* expression cannot be explained by these changes.

### MAL-Dependent Differential Gene Expression in Schwann Cells

To further investigate the effect of MAL overexpression on gene transcription, microarray data were analyzed more stringently using an FDR-adjusted *p* value of < 0.05. This study revealed that the mRNA expression levels of 15 genes were highly significantly reduced in MAL-overexpressing Schwann cells, whereas those of 7 genes were increased (Table 2). Literature search revealed that the expression of most of these genes has not yet been reported in Schwann cells.

**Table 2.** Highly Significantly Changed Gene Expression due to MAL Overexpression.

Common name	Entrez ID	Putative biological function	Role in Schwann cells	tgMAL:wt	*p*
Decreased due to MAL overexpression					
Glutamic acid decarboxylase 2 (Gad65)	***Gad2***	Decarboxylates glutamate to vesicular GABA	Detected in sciatic nerve extracts (Magnaghi et al., 2010)	0.587	<0.00001
Asialoglycoprotein receptor 1	***Asgr1***	Mediates endocytosis of plasma glycoproteins in hepatocytes; turnover of glycoproteins (Rigopoulou et al., 2012)	not reported	0.605	<0.00001
Ectonucleoside triphosphate diphosphohydrolase 2 (NTPDase2)	***Entpd2***	Hydrolyzes extracellular ATP to ADP	Expressed in immature and nonmyelinating Schwann cells (Braun et al., 2004)	0.622	0.00002
Keratin 23	***Krt23***	Intermediate filament for structural integrity in epithelial cells (Zhang et al., 2001)	not reported	0.643	0.00006
Synaptic vesicle glycoprotein 2 b	***Sv2b***	Integral membrane protein, essential for normal neurotransmission, proteoglycan at ECM (Scranton et al., 1993; Sinouris et al., 2009)	Expressed on axonal side at node of Ranvier (Zimmermann, 1996)	0.669	0.00004
TRAF2 and NCK interacting kinase	***Tnik***	Regulates cytoskeleton by interaction with F-actin, activates JNK pathway (Fu et al., 1999)	not reported	0.676 0.829	<0.00001 <0.00001
DIRAS family, GTP-binding RAS-like 1	***Diras1***	Member of the small GTPase Ras family with only low GTPase activity, implicated in protein transport and localization (Kontani et al., 2002)	not reported	0.693	<0.00001
Chloride channel calcium activated 4	***Clca4***	N-terminal domain suggested to act as a zinc metalloprotease	not reported	0.729	0.00004
Vesicle amine transport protein 1 homolog-like	***Vat1l***	Zinc-containing alcohol dehydrogenase family	not reported	0.752	0.00001
Immunoglobulin superfamily, member 10	***Igsf10***	Modulated by ECM in colorectal cancer cells (Zvibel et al., 2013)	not reported	0.757 0.767	<0.00001 <0.00001
6-phosphofructo-2-kinase/fructose-2,6-biphosphatase 4	***Pfkfb4***	Regulates concentration of fructose 2,6-bisphosphate	Glycolysis present in Schwann cells	0.773	0.00004
Family with sequence similarity 213, member B	***Fam213b***	–	not reported	0.776	0.00006
Glutamate receptor, ionotropic, AMPA1 (Gria, GluR-A, Glur1)	***Gria1***	Neurotransmission	Detected in a microarray study on human Schwann cell culture (Lee et al., 2004)	0.783 0.818	0.00001 0.00002
Ras homolog family member U (Wrch1)	***Rhou***	Member of Cdc42-related subfamily, induces actin reorganization and filopodia formation, required for F-actin polarization in the endoderm, implicated in activation of AKT and JNK (Chuang et al., 2007; Feltri et al., 2008; Loebel et al., 2011; Ory et al., 2007; Tao et al., 2001)	not reported	0.793	<0.00001
α-Dystrobrevin	***Dtna***	Member of dystrophin-glycoprotein complex (links extracellular matrix to cytoskeleton)	Expressed in perineurium and Schwann cells (Albrecht et al., 2008)	0.799	0.00001
Increased due to MAL overexpression					
Myelin and lymphocyte protein	***Mal***	Localized in lipid raft, trafficking & signaling; nervous system, epithelial cells	Overexpression leads to delayed onset of myelination, reduced p75^NTR^ expression and altered Remak bundle formation (Buser et al., 2009b)	21.533 12.687 1.168	<0.00001 <0.00001 0.00006
Monooxygenase, DBH-like 1	***Moxd1***	Predicted to hydroxylate a hydrophobic substrate in the endoplasmic reticulum	not reported	1.564 1.382	<0.00001 <0.00001
Wingless-related MMTV integration site 16	***Wnt16***	Secreted signaling protein, Wnt16B activates JNK pathway, regulates PI3-Kinase AKT pathway (Binet et al, 2009; Teh et al., 2007)	Wnt/beta-Catenin Signaling is important for expression of myelin genes (Tawk et al., 2011)	1.380	0.00007
S100 calcium binding protein A4 (Mts1)	***S100a4***	Ca2+-binding protein in tumor metastasis, regulates cytoskeleton by binding to tropomyosin and non-muscle myosin II (Li et al., 2003; Watanabe et al., 1993)	Increased upon PMP22 overexpression (ten Asbroek et al., 2005) and upon nerve injury (Sandelin et al., 2004); suggested as neuroprotectant (Moldovan et al., 2013)	1.279	0.00002
Oxysterol binding protein-like 3	***Osbpl3***	Intracellular lipid receptors. OSBP as sterol sensor; sterol-dependent scaffold for ERK pathway regulation	Oxysterol present in Schwann cells, inhibits myelin gene expression (Makoukji et al., 2011)	1.270	<0.00001
Aquaporin 1	***Aqp1***	Water channel	Localized in peripheral nerves (Gao et al., 2006)	1.266	0.00005
LIM domain binding 2	***Ldb2***	Regulation of cell migration, biosensor that mediates communication between cytosolic and nuclear compartments (Storbeck et al., 2009)	not reported	1.235	0.00003

*Note.* A whole genome expression analysis was investigated in MAL-overexpressing and wild-type Schwann cells. Data were analyzed using a three-way analysis of variance with an FDR-adjusted *p* value of <0.05, and transcripts with at least 20% expressional changes were taken into account. The mRNA expression levels of 15 gene transcripts were decreased, whereas those of 7 were increased in MAL-overexpressing Schwann cells.

In MAL-overexpressing Schwann cells, substantial transcriptional reduction was detected for the glutamic acid decarboxylase 2 (*Gad2*/*Gad65*), the asialoglycoprotein receptor 1 (*Asgr1*), the ectonucleoside triphosphate diphosphohydrolase 2 (*Entpd2*), and the intermediate filament keratin 23 (*Krt23*). MAL overexpression resulted also in reduced mRNA expression levels of the proteoglycan synaptic vesicle glycoprotein 2B (*Sv2b*), the TRAF2 and NCK interacting kinase (*Tnik*), the GTP-binding protein Di-Ras1 (*Diras1*), and the chloride channel calcium-activated 4 (*Clca4*). In addition, significant reduction of mRNA expression levels could be detected for the vesicle amine transport protein 1 homolog-like (*Vat1l*), the member 10 of the immunoglobulin superfamily (*Igsf10*), as well as the glycolysis-influencing gene 6-phosphofructo-2-kinase/fructose-2,6-biphosphatase 4 (*Pfkfb4*). A comparable reduction was identified for the ionotropic glutamate receptor AMPA1 (*Gria1*/*GluR-A*/*Glur1*), the ras homolog family member U (*RhoU*/*Wrch1*), and α-dystrobrevin (*Dtna*).

In MAL-overexpressing Schwann cells, significantly increased expression levels of *Mal* were detected, serving as a positive control. Further, increased mRNA expression levels were observed for the monooxygenase DBH-like 1 (*Moxd1*), the secreted signaling protein *Wnt16*, as well as the tropomyosin- and nonmuscle myosin II-binding protein *S100a4*/*Mts1*. Upon MAL overexpression, the intracellular lipid receptor oxysterol-binding protein-like 3 (*Osbpl3*), the water channel aquaporin 1 (*Aqp1*), and the transcriptional cofactor LIM domain-binding 2 (*Ldb2*) were also identified to be significantly increased.

To detect putative functional clustering of the MAL-dependent differentially expressed genes, a hierarchical cluster analysis was performed (Supplementary Figure 3). This analysis revealed an unambiguous separation between untreated and treated Schwann cells, indicating that the hierarchical clusters of genes were primarily influenced by their expression changes upon forskolin treatment. Further, most of the samples of the respective genotype clustered within the same stimulation condition. However, a functional clustering of particular gene sequences was not evident.

### Investigation of Differentially Expressed Genes due to MAL Overexpression In Vivo

**Figure 3.**
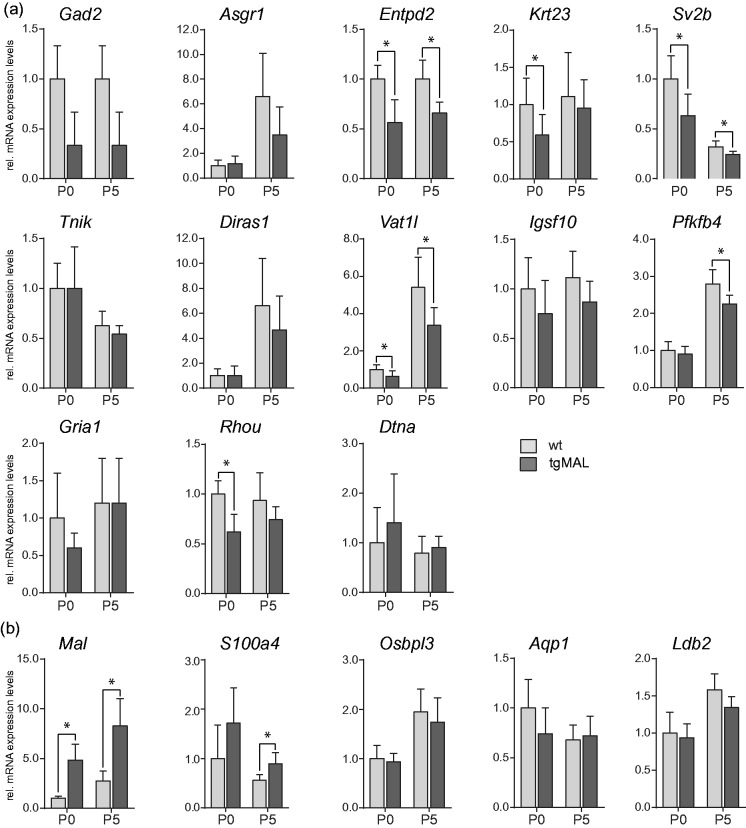
Investigation of transcriptional expression in MAL-overexpressing and wild-type sciatic nerves. Gene transcripts significantly reduced (a) or increased (b) in MAL-overexpressing Schwann cells were analyzed in P0 and P5 sciatic nerves of MAL-overexpressing mice (tgMAL) and wild-type littermates (wt). *Entpd2*, *Krt23*, *Sv2b*, *Vat1l*, *Pfkfb4*, and *Rhou* were significantly decreased in mice overexpressing MAL (a), whereas the expression levels of *Mal* and *S100a4* were significantly increased (b). Data were normalized to the expression of *60s*, and P0 wild-type values were set to 1. The columns show the mean value of at least four experimental samples, and the error bars indicate the *SD*. *p ≤ 0.05.

The expression pattern of the newly identified differentially expressed transcripts in Schwann cells overexpressing MAL was further investigated in sciatic nerves of MAL-overexpressing mice and wild-type littermates at birth and at P5 by qRT-PCR (Figure 3, Supplementary Table 3). Significantly reduced mRNA expression levels were detected for *Entpd2*, *Krt23*, *Sv2b*, *Vat1l*, *Pfkfb4*, and *Rhou* [Figure 3(a)], which is in line with the *in vitro* situation. Reduced gene transcription could also be observed for the other genes although not significant (*p* > 0.05) due to their higher variable expression levels at these early developmental time points. The only exception was for *Tnik* and *Dtna*, for which no differential expression was observed in peripheral nerve tissue neither at early nor at later developmental time points (P10, P20, adult, data not shown). As expected, the strong induction of *Mal* mRNA levels could be confirmed in MAL-overexpressing nerves at both investigated time points [Figure 3(b)]. This was also the case for *S100a4* mRNA expression that was significantly increased in MAL-overexpressing nerves, in line with the *in vitro* observation. In comparison to primary mouse Schwann cell cultures, no differential expression of *Osbpl3*, *Aqp1*, and *Ldb2* was detected *in vivo*, and *Clca4*, *Moxd1*, and *Wnt16* were expressed below background levels. These results demonstrate that MAL-overexpression influences transcriptional regulation of most of the newly identified genes both *in vitro* and *in vivo*, suggesting a functional relevance in Schwann cells.

### S100a4 Is Predominantly Expressed in Nonmyelinating Schwann Cells

**Figure 4.**
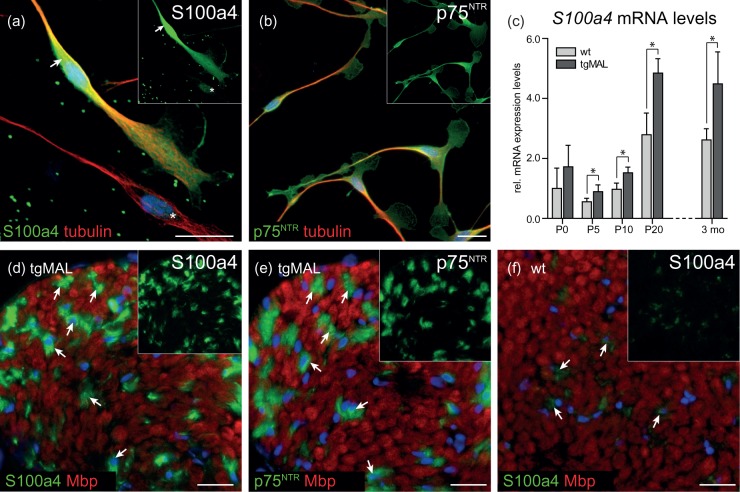
Investigation of the spatial expression pattern of S100a4 in cultured Schwann cells and in sciatic nerves. Immunofluorescent microscopy revealed a strong cytoplasmic S100a4 expression in a subpopulation of cultured Schwann cells (a, arrow). Due to this strong immunofluorescent signal, the acquisition time was reduced, and therefore, cells still considerably expressing S100a4 appeared only weakly positive (a, asterisk). (b) In contrast to S100a4, p75^NTR^ signal was observed at a homogenous intensity level, indicating that those cells belong to the Schwann cell lineage. (c) Analysis of *S100a4* mRNA levels revealed robustly increased expression levels in sciatic nerves derived from MAL-overexpressing mice at all investigated time points. Data were normalized to the expression of *60s*, and P0 wild-type values were set to 1. The columns show the mean value of at least four experimental samples, and the error bars indicate the *SD*. * *p* ≤ 0.05. Please note that data for P0 and P5 were already presented in Figure 3. (d) Spatial expression analysis on sciatic nerves of 3-month-old MAL-overexpressing mice revealed strong S100a4 signal in Remak bundles, identified as Mbp-negative areas (d, arrows). (e) The same areas were also positive for p75^NTR^, a marker for nonmyelinating Schwann cells (e, arrows). (f) Only weak expression of S100a4 was detected in wild-type sciatic nerves; its localization was comparable to MAL-overexpressing mice (f, arrows). Blue: DAPI. Bars: 20 µm.

Cytoskeletal dynamics is crucial for Schwann cell development. For S100a4, which regulates the cytoskeleton organization by promoting the disassembly of myosin II filaments (Li et al., 2003), significantly increased mRNA expression levels were observed upon MAL overexpression both *in vivo* and *in vitro*. For this reason, the spatial protein expression pattern of S100a4 was investigated (Figure 4). In primary Schwann cell cultures, immunofluorescent microscopy revealed that all Schwann cells, identified by p75^NTR^ [Figure 4(b)], expressed S100a4, but a subpopulation of Schwann cells showed very high expression of S100a4 in the cytoplasm [Figure 4(a), arrow].

We further investigated the expression of *S100a4* *in vivo*. In addition to P0 and P5, significantly increased mRNA expression levels of *S100a4* were also detected at P10, P20, and in adult mice overexpressing MAL [Figure 4(c)]. The spatial expression pattern of S100a4 was also investigated on transversal sciatic nerve sections of 3-month-old MAL-overexpressing and wild-type mice. We detected a strong S100a4 immunofluorescent signal in Remak bundles in MAL-overexpressing mice, identified as Mbp-negative areas [Figure 4(d), arrows]. Remak bundles in MAL-overexpressing nerves are readily identifiable due to progressive segregation of unmyelinated axons observed in these mice (Buser et al., 2009b) and due to expression of p75^NTR^, a marker for nonmyelinating Schwann cells [Figure 4(e), arrows]. In wild-type mice, only weak S100a4 immunofluorescent signal was detected in Remak bundles, confirming increased S100a4 expression in MAL-overexpressing mice not only on mRNA but also on protein levels [Figure 4(f), arrows]. In summary, our data show that the expression levels of S100a4 mRNA and protein, a regulator of the cytoskeleton organization, were upregulated upon MAL overexpression *in vitro* and *in vivo*. Further, we detected S100a4 protein to be predominantly expressed in nonmyelinating Schwann cells.

### Cellular Expression Pattern of RhoU and Krt23 in Schwann Cells

**Figure 5.**
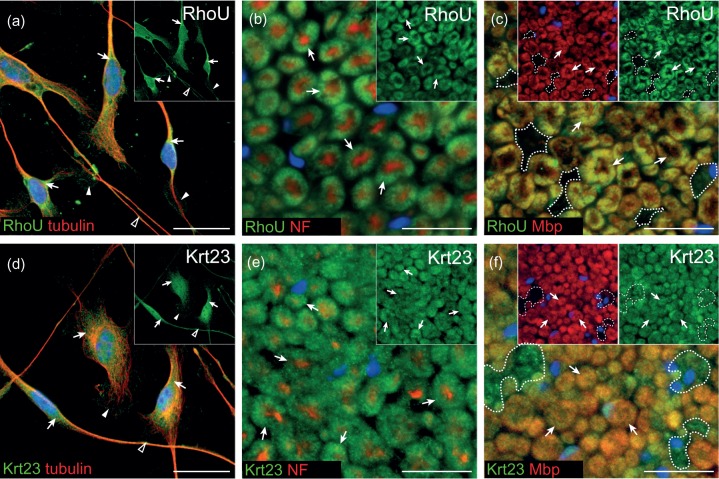
Localization of RhoU and Krt23 in cultured Schwann cells and in sciatic nerves. (a) In primary mouse Schwann cell cultures, immunofluorescent signal for RhoU was detected within the cytoplasm (a, arrows), in membrane protrusions (a, arrowheads), and in Schwann cell processes (A, open arrowhead). (b, c) In sciatic nerves of 3-month-old mice, RhoU was expressed in myelinating Schwann cells (B, C, arrows), whereas nonmyelinating Schwann cells in Mbp-negative area did not express RhoU (c, encircled area). (d) For Krt23, a punctated staining pattern was present in the cytoplasm of cultured Schwann cells (d, arrows). Its localization was further detected in membrane protrusions (d, arrowhead) as well as along Schwann cell processes (d, open arrowhead). (e, f) Immunofluorescent microscopy on sciatic nerves of 3-month-old mice revealed Krt23 to be punctately localized in myelinating Schwann cells (e, f, arrows), as well as in nonmyelinating Schwann cells (f, encircled area). Localization in nonmyelinating Schwann cells was visualized on sciatic nerves derived from MAL-overexpressing mice (c, f). Blue: DAPI. Bars: 20 µm.

We further investigated the expression pattern of RhoU and Krt23, which are also known to be involved in cytoskeleton organization, and were significantly reduced by MAL overexpression both in cultured Schwann cells and in sciatic nerves of newborn mice. First, their spatial localization was investigated in cultured primary mouse Schwann cells. The Cdc42-related superfamily member RhoU was localized throughout the cytoplasm [Figure 5(a), arrows], within membrane protrusions [Figure 5(a), arrowheads] as well as along Schwann cell processes [Figure 5(a), open arrowhead]. Immunofluorescent microscopy for the intermediate filament Krt23 detected a punctated staining pattern in the cytoplasm [Figure 5(d), arrows] and in membrane protrusions [Figure 5(d), arrowhead]. Further, Krt23 immunofluorescence could be identified within and along Schwann cell processes [Figure 5(d), open arrowhead]. In addition to cultured Schwann cells, the expression pattern of RhoU and Krt23 was investigated on transversal sciatic nerve sections of adult mice [Figure 5(b), (c), (e), and (f)]. RhoU protein was detected within the myelin sheath [Figure 5(b), arrows], identified by Mbp [Figure 5(c), arrows], but not in nonmyelinating Schwann cells in Remak bundles [Figure 5(c), encircled area]. For Krt23, a punctated staining was observed in myelin membranes [Figure 5(e) and (f), arrows] as well as in nonmyelinating Schwann cells [Figure 5(f), encircled area].

Our data show that RhoU was localized within myelin membranes, and Krt23 was detected in nonmyelinating as well as in myelinating Schwann cells, indicating that overexpression of MAL influences both nonmyelinating and myelinating Schwann cells.

### Analysis of Annotation Tags for Significantly Altered Genes in MAL-Overexpressing Schwann Cell Cultures

A possible common functional role of the 15 genes, whose transcripts were highly significantly decreased in MAL-overexpressing Schwann cells, was investigated by the IPA of Ingenuity Systems and the DAVID. No common signaling pathway, protein–protein interactions, or common transcriptional regulator for those genes was identified. However, functional annotation charts revealed that almost one third of the investigated genes (*Asgr1*, *Gria1*, *Gad2*, and *Rhou*) were associated with the term palmitate (*p* < 0.0005, indicating that these genes are specifically associated with this term more often than would be expected by chance). All of them were also reduced *in vivo*, suggesting a functional role in Schwann cells. Palmitoylation has been shown to be important for efficient targeting of myelin proteins to the plasma membrane (Schneider et al., 2005). These data support the current concept for the functional role of MAL in sorting and trafficking of particular components to the plasma membrane (Schaeren-Wiemers et al., 2004).

## Discussion

A previous study on PNS development revealed that MAL overexpression led to a delayed onset of myelination, hypomyelinated fibers, and reduced *Mpz* and *p75^NTR^* expression levels in newborn mice (Buser et al., 2009b). We postulated that MAL plays a critical role in PNS myelin formation, probably by influencing the trafficking of particular membrane components important for axon-glia interaction and downstream signaling during the process of myelin initiation. From that study, we concluded that MAL dosage influences *Mpz* and *p75^NTR^* expression levels in the PNS and maybe by that the progress of myelination. This is unexpected because MAL is considered to be a regulator of raft-dependent protein transport processes but not a regulator of gene expression. However, our data point to a cascade of events that ultimately leads to reduced expression levels of the major myelin protein *Mpz* and the *p75^NTR^* receptor and to delayed myelination in MAL-overexpressing mice. In this study, the consequence of MAL overexpression was investigated in primary mouse Schwann cell cultures. We analyzed its effect on the transcriptional level as well as its influence on major signaling pathways known to be important for Schwann cell differentiation and myelination. Significantly reduced mRNA expression levels were detected for *Mpz* and *p75^NTR^* in cultured MAL-overexpressing Schwann cells, validating the *in vivo* observation. Reduced expression of *Mpz* and *p75^NTR^* was already evident before Schwann cell differentiation, implicating that the effect of MAL overexpression affects early processes in Schwann cell development. Herein this study, we further demonstrated that primary mouse Schwann cells overexpressing MAL can activate the CREB signaling pathway upon treatment with forskolin, indicated by a comparable induction of *Mpz* expression as observed in wild-type cells. We also showed that the delayed onset of myelination *in vivo* is not due to impaired phosphorylation of Akt, suggesting that the PI3-kinase pathway is functional. In line, phosphorylation of Akt was induced in MAL-overexpressing Schwann cell cultures in a comparable degree as in wild-type cells. Differential expression analysis of genes implicated to be regulated upon activation of the Raf-kinase (Napoli et al., 2012) revealed no transcriptional alterations in MAL-overexpressing Schwann cells, also demonstrating that the ERK pathway seems not to be affected by MAL overexpression (Supplementary Table 4).

### MAL Overexpression Leads to Differential Expression of Genes Associated With Cytoskeleton Organization and ECM

Despite the fact that *Krox20* mRNA was increased by qRT-PCR, the expression levels of *Mpz* and *p75^NTR^* were robustly decreased. Hence, the consistent reduction of *Mpz* and *p75^NTR^* expression in MAL-overexpressing Schwann cells cannot be explained by the minor transcriptional effects of known genes in Schwann cells. For this reason, a whole genome microarray was performed, and a set of genes implicated in the regulation of the cytoskeleton was identified to be differentially regulated by MAL overexpression [Supplementary Figure 4(a)]. One differentially expressed gene transcript was *S100a4*, a member of the EF-hand family of calcium-binding protein. In general, S100a4 inhibits the assembly of nonmuscle myosin II monomers into filaments and promotes the disassembly of myosin II filaments, implicated in remodeling the actin cytoskeleton [Li et al., 2003, reviewed in Vicente-Manzanares et al., 2009; Supplementary Figure 4(b)]. In Schwann cells, myosin II was shown to be necessary for peripheral myelination, indicated by reduced number of myelin segments, reduced expression of myelin proteins, as well as impaired basal lamina assembly shown by RNA interference in myelinating cocultures (Wang et al., 2008). Our study revealed that MAL overexpression led to increased mRNA and protein levels of S100a4 both *in vitro* and *in vivo* throughout development and in the adult. Increased *S100a4* expression in MAL-overexpressing Schwann cells might therefore increase the disassembly of myosin II filaments, thereby influencing actin filament stability. Further, immunofluorescent colocalization analysis in peripheral nerves identified S100a4 to be expressed predominantely in nonmyelinating Schwann cells. This observation suggests that the strongly S100a4 positive Schwann cells *in vitro* belong to the nonmyelinating Schwann cell lineage.

Another gene associated with cytoskeleton organization is the intermediate filament keratin 23 (*Krt23*), a member of the type I cytokeratin family (Zhang et al., 2001). The expression of *Krt23* mRNA was strongly reduced upon MAL overexpression both *in vitro* and *in vivo*. Immunofluorescent microscopy of adult peripheral nerves revealed its protein localization in myelinating as well as in nonmyelinating Schwann cells. For Krt18, an interaction partner of Krt23 (Liffers et al., 2011), keratin monomers were shown to appear at the cell periphery close to the plasma membrane, and to be transported along actin fibers, allowing intermediate filament network reorganization [Kolsch et al., 2009; Supplementary Figure 4(b)]. In Schwann cells, only little is known about the expression of keratins. Our previous analysis revealed that *Krt23* and *Krt10* were substantially expressed in Schwann cells *in vitro*, whereas other keratin transcripts were not detected in primary Schwann cell cultures (Schmid et al., 2014). Thus, reduced expression of *Krt23* in MAL-overexpressing Schwann cells might debilitate integration of keratin monomers into the keratin filament network that is mediated by actin fibers.

Along these lines, we identified reduced expression of *RhoU*/*Wrch1* in cultured MAL-overexpressing Schwann cells and in sciatic nerves of newborn MAL-overexpressing mice. Spatial expression analysis on sciatic nerves revealed that RhoU is expressed by myelinating but not by nonmyelinating Schwann cells. RhoU is implicated in the activation of the JNK signaling pathway and is essential to maintain F-actin polarization [Ory et al., 2007; Tao et al., 2001; Supplementary Figure 4(b)]. RhoU overexpression results in altered cytoskeletal architecture by dissolution of F-actin stress fibers in 3T3 cells (Tao et al., 2001). In MAL-overexpressing Schwann cells, we further observed a decreased mRNA expression levels of the TRAF2 and NCK-interacting kinase (*Tnik*), belonging to the germinal center kinase (GCK) family [Supplementary Figure 4(b)]. Tnik specifically activates the JNK pathway and induces disruption of F-actin and by that inhibits cell spreading (Fu et al., 1999). However, reduced expression could not be observed in sciatic nerve tissues derived from MAL-overexpressing mice, suggesting that MAL overexpression does not have a general impact on the expression of *Tnik*. Its expression might be tightly regulated within a particular stage during Schwann cell development or differentiation.

The observed reduced expression of *Tnik* and *RhoU* in MAL-overexpressing Schwann cells may influence their potential to induce disassembly of F-actin and by that to modulate cytoskeleton dynamics important for Schwann cell membrane mobility. Their functional role in Schwann cells is not yet known, but they might be novel candidates involved in modulating early events of Schwann cell differentiation, in which JNK and actin are known to play an important role (Fernandez-Valle et al., 1997; Parkinson et al., 2004). In line, the reduced expression of *Cnp* in MAL-overexpressing Schwann cells may also point to altered cytoskeletal dynamics. Cnp was shown to bind to tubulin heterodimers and to influence F-actin and microtubule reorganization in oligodendrocytes (Lee et al. 2005). In a previous study, we reported reduced protein expression of Cnp in membrane preparations of P5 sciatic nerves of MAL-overexpressing mice (Buser et al., 2009b). Hitherto, it was unknown whether this reduced expression is a consequence of the delayed onset of myelination or whether it is directly linked to MAL overexpression. Now we can speculate that reduced Cnp protein levels *in vivo* are a direct consequence of MAL overexpression and not of the delayed myelination, because other myelin-associated genes were not differentially regulated in MAL-overexpressing Schwann cell cultures.

Besides microtubules, actin filaments, and intermediate filaments, a fourth filamentous system is known in Schwann cells, namely, the family of septins (Buser et al., 2009a). Septins form higher order filaments by heteromeric assembly and can interact with components of cellular membranes, as well as with actin filaments and microtubules (reviewed in Saarikangas & Barral, 2011). In polarized cells, septins are involved in membrane compartmentalization and vesicle transport. A whole set of septins are expressed in Schwann cells throughout development and in the adult, and they were localized in particular cellular compartments (Buser et al., 2009a). MAL was shown to interact with septin 6, which in turn is associated with particular septins and actin, probably establishing specialized scaffolds in distinct myelin compartments [Buser et al., 2009a; Supplementary Figure 4(c)]. Due to this direct link between MAL and a component of the cytoskeleton, it is tempting to speculate about the modulatory role of MAL in plasma membrane mobility.

During Schwann cell development and differentiation, accurate cytoskeleton organization is crucial, and altered cytoskeletal architecture might influence the onset of myelination by disturbing radial sorting. Two important modulators for radial sorting are the Rho family GTPases *Rac1* and *Cdc42* (Benninger et al., 2007; Nodari et al., 2007). Analysis of downstream targets of the *Rac1* and *Cdc42* signaling pathways revealed, however, no altered transcription in MAL-overexpressing Schwann cells, proposing that these signaling cascades are also functional in MAL-overexpressing Schwann cells (Supplementary Table 5).

Besides proper cytoskeleton organization, the correct assembly of the ECM is also essential for accurate myelin formation. Indeed, a number of transcripts of genes associated with the ECM were differentially regulated in Schwann cells overexpressing MAL, such as *α-dystrobrevin*, *dystroglycan*, and *collagen type II α1*. In addition to proper gene expression levels of ECM members, accurate sulfatide levels are required to anchor laminin-1 (α1β1γ1) and laminin-2 (α2β1γ1) to Schwann cells and consequently to assemble the basal lamina (Li et al., 2005; McKee et al., 2007). Interestingly, previous reports showed that MAL binds sulfatide (Frank et al., 1998; Saravanan et al., 2004). This finding suggests that MAL-dependent binding of sulfatide influences functional integration of laminins into the basal lamina, resulting in impaired integrin- and dystroglycan-mediated signaling and by that effect myelination in Schwann cells.

### MAL Overexpression Altered Expression of Genes Associated With Palmitate

Bioinformatic analysis of highly significantly reduced genes in MAL-overexpressing Schwann cells revealed an enrichment of the term palmitate. Palmitoylation is a reversible posttranslational modification by covalently adding the saturated fatty acid palmitate to cysteine residues via thioester linkages, leading to increased protein hydrophobicity, and consequently facilitates interaction with lipid bilayer (reviewed in Linder & Deschenes, 2007). Long-chain saturated-free fatty acids such as palmitate accumulate in membrane lipid rafts and influence the lipid environment, which might result in altered downstream signaling by affecting receptor functions (reviewed in Simons & Toomre, 2000). In Schwann cells, palmitoylation is required to translocate p75^NTR^ protein into cholesterol-rich domains of the plasma membrane, which is crucial for subsequent cleavage by the γ-secretase (Underwood et al., 2008). Further, palmitoylation of Pmp22 and Plp is implicated in the efficient protein transport to the plasma membrane (Schneider et al., 2005; Zoltewicz et al., 2012). The protein MAL is an important component of lipid rafts and is localized in the trans-Golgi network, where lipid rafts are constituted. It is tempting to speculate that MAL overexpression alters the composition of lipid rafts, consequently disturbing the balance of accurate palmitoylated proteins in particular signaling platforms. Thus, altered palmitoylation might lead to impaired downstream signaling influencing the efficiency of myelination in newborn MAL-overexpressing mice.

## Conclusion

MAL overexpression manifests retarted maturation of nonmyelinating and myelinating Schwann cells, indicated by differential expression of the myelin protein *Mpz* and the neurotrophin receptor *p75^NTR^* (Buser et al., 2009b). This presented study describes novel genes expressed by nonmyelinating as well as myelinating Schwann cells, which are differentially expressed in a MAL-dependent manner and are implicated in the regulation of cytoskeletal organization and plasma membrane mobility.

## Summary statement

MAL overexpression leads to reduced expression of *Mpz* and *p75^NTR^*, despite functional pathways and normal expression of genes important for Schwann cell differentiation. Particular differentially expressed genes implicated in cytoskeleton organization were identified, proposing them as novel candidates influencing myelination.

## References

[bibr1-1759091414548916] Albrecht D. E. Sherman D. L. Brophy P. J. Froehner S. C (2008) The ABCA1 cholesterol transporter associates with one of two distinct dystrophin-based scaffolds in Schwann cells. Glia 56: 611–618. 18286648 10.1002/glia.20636 PMC4335170

[bibr2-1759091414548916] Benjamini Y. Hochberg Y (1995) Controlling the false discovery rate: A practical and powerful approach to multiple testing. Journal of the Royal Statistical Society: Series B 57: 289–300.

[bibr3-1759091414548916] Benninger Y. Thurnherr T. Pereira J. A. Krause S. Wu X. Chrostek-Grashoff A. Relvas J. B (2007) Essential and distinct roles for cdc42 and rac1 in the regulation of Schwann cell biology during peripheral nervous system development. The Journal of Cell Biology 177: 1051–1061. 17576798 10.1083/jcb.200610108 PMC2064365

[bibr4-1759091414548916] Bermingham J. R. Jr Scherer S. S. O'Connell S. Arroyo E. Kalla K. A. Powell F. L. Rosenfeld M. G (1996) Tst-1/Oct-6/SCIP regulates a unique step in peripheral myelination and is required for normal respiration. Genes & Development 10: 1751–1762. 8698235 10.1101/gad.10.14.1751

[bibr5-1759091414548916] Binet R. Ythier D. Robles A. I. Collado M. Larrieu D. Fonti C. Pedeux R (2009) WNT16B is a new marker of cellular senescence that regulates p53 activity and the phosphoinositide 3-kinase/AKT pathway. Cancer Research 69: 9183–9191. 19951988 10.1158/0008-5472.CAN-09-1016 PMC7439003

[bibr6-1759091414548916] Bosse F. Hasse B. Pippirs U. Greiner-Petter R. Muller H. W (2003) Proteolipid plasmolipin: Localization in polarized cells, regulated expression and lipid raft association in CNS and PNS myelin. Journal of Neurochemistry 86: 508–518. 12871592 10.1046/j.1471-4159.2003.01870.x

[bibr7-1759091414548916] Braun N. Sevigny J. Robson S. C. Hammer K. Hanani M. Zimmermann H (2004) Association of the ecto-ATPase NTPDase2 with glial cells of the peripheral nervous system. Glia 45: 124–132. 14730706 10.1002/glia.10309

[bibr8-1759091414548916] Buser A. M. Erne B. Werner H. B. Nave K. A. Schaeren-Wiemers N (2009a) The septin cytoskeleton in myelinating glia. Molecular and Cellular Neurosciences 40: 156–166. 19026747 10.1016/j.mcn.2008.10.002

[bibr9-1759091414548916] Buser A. M. Schmid D. Kern F. Erne B. Lazzati T. Schaeren-Wiemers N (2009b) The myelin protein MAL affects peripheral nerve myelination: A new player influencing p75 neurotrophin receptor expression. The European Journal of Neuroscience 29: 2276–2290. 19508690 10.1111/j.1460-9568.2009.06785.x

[bibr10-1759091414548916] Chuang Y. Y. Valster A. Coniglio S. J. Backer J. M. Symons M (2007) The atypical Rho family GTPase Wrch-1 regulates focal adhesion formation and cell migration. Journal of Cell Science 120: 1927–1934. 17504809 10.1242/jcs.03456

[bibr11-1759091414548916] Cochary E. F. Bizzozero O. A. Sapirstein V. S. Nolan C. E. Fischer I (1990) Presence of the plasma membrane proteolipid (plasmolipin) in myelin. Journal of Neurochemistry 55: 602–610. 1695242 10.1111/j.1471-4159.1990.tb04176.x

[bibr12-1759091414548916] Cosgaya J. M. Chan J. R. Shooter E. M (2002) The neurotrophin receptor p75NTR as a positive modulator of myelination. Science 298: 1245–1248. 12424382 10.1126/science.1076595

[bibr13-1759091414548916] Erne B. Sansano S. Frank M. Schaeren-Wiemers N (2002) Rafts in adult peripheral nerve myelin contain major structural myelin proteins and myelin and lymphocyte protein (MAL) and CD59 as specific markers. Journal of Neurochemistry 82: 550–562. 12153479 10.1046/j.1471-4159.2002.00987.x

[bibr14-1759091414548916] Feltri M. L. Suter U. Relvas J. B (2008) The function of RhoGTPases in axon ensheathment and myelination. Glia 56: 1508–1517. 18803320 10.1002/glia.20752 PMC2615182

[bibr15-1759091414548916] Fernandez-Valle C. Gorman D. Gomez A. M. Bunge M. B (1997) Actin plays a role in both changes in cell shape and gene-expression associated with Schwann cell myelination. The Journal of Neuroscience: The Official Journal of the Society for Neuroscience 17: 241–250. 8987752 10.1523/JNEUROSCI.17-01-00241.1997 PMC6793673

[bibr16-1759091414548916] Frank M (2000) MAL, a proteolipid in glycosphingolipid enriched domains: Functional implications in myelin and beyond. Progress in Neurobiology 60: 531–544. 10739088 10.1016/s0301-0082(99)00039-8

[bibr17-1759091414548916] Frank M. Atanasoski S. Sancho S. Magyar J. P. Rulicke T. Schwab M. E. Suter U (2000) Progressive segregation of unmyelinated axons in peripheral nerves, myelin alterations in the CNS, and cyst formation in the kidneys of myelin and lymphocyte protein-overexpressing mice. Journal of Neurochemistry 75: 1927–1939. 11032882 10.1046/j.1471-4159.2000.0751927.x

[bibr18-1759091414548916] Frank M. Schaeren-Wiemers N. Schneider R. Schwab M. E (1999) Developmental expression pattern of the myelin proteolipid MAL indicates different functions of MAL for immature Schwann cells and in a late step of CNS myelinogenesis. Journal of Neurochemistry 73: 587–597. 10428054 10.1046/j.1471-4159.1999.0730587.x

[bibr19-1759091414548916] Frank M. van der Haar M. E. Schaeren-Wiemers N. Schwab M. E (1998) rMAL is a glycosphingolipid-associated protein of myelin and apical membranes of epithelial cells in kidney and stomach. The Journal of Neuroscience: The Official Journal of the Society for Neuroscience 18: 4901–4913. 9634556 10.1523/JNEUROSCI.18-13-04901.1998 PMC6792556

[bibr20-1759091414548916] Fu C. A. Shen M. Huang B. C. Lasaga J. Payan D. G. Luo Y (1999) TNIK, a novel member of the germinal center kinase family that activates the c-Jun N-terminal kinase pathway and regulates the cytoskeleton. The Journal of Biological Chemistry 274: 30729–30737. 10521462 10.1074/jbc.274.43.30729

[bibr21-1759091414548916] Gao H. He C. Fang X. Hou X. Feng X. Yang H. Ma T (2006) Localization of aquaporin-1 water channel in glial cells of the human peripheral nervous system. Glia 53: 783–787. 16534779 10.1002/glia.20336

[bibr22-1759091414548916] Gao X. Daugherty R. L. Tourtellotte W. G (2007) Regulation of low affinity neurotrophin receptor (p75(NTR)) by early growth response (Egr) transcriptional regulators. Molecular and Cellular Neurosciences 36: 501–514. 17916431 10.1016/j.mcn.2007.08.013 PMC2098703

[bibr23-1759091414548916] Garratt A. N. Britsch S. Birchmeier C (2000) Neuregulin, a factor with many functions in the life of a schwann cell. BioEssays: News and Reviews in Molecular, Cellular and Developmental Biology 22: 987–996. 10.1002/1521-1878(200011)22:11<987::AID-BIES5>3.0.CO;2-5 11056475

[bibr24-1759091414548916] Huang da W. Sherman B. T. Lempicki R. A (2009) Systematic and integrative analysis of large gene lists using DAVID bioinformatics resources. Nature Protocols 4: 44–57. 10.1038/nprot.2008.211 19131956

[bibr25-1759091414548916] Jaegle M. Mandemakers W. Broos L. Zwart R. Karis A. Visser P. Meijer D (1996) The POU factor Oct-6 and Schwann cell differentiation. Science 273: 507–510. 8662541 10.1126/science.273.5274.507

[bibr26-1759091414548916] Kinter J. Lazzati T. Schmid D. Zeis T. Erne B. Lutzelschwab R. Schaeren-Wiemers N (2013) An essential role of MAG in mediating axon-myelin attachment in Charcot-Marie-Tooth 1A disease. Neurobiology of Disease 49: 221–231. 22940629 10.1016/j.nbd.2012.08.009 PMC3612363

[bibr27-1759091414548916] Kolsch A. Windoffer R. Leube R. E (2009) Actin-dependent dynamics of keratin filament precursors. Cell Motility and the Cytoskeleton 66: 976–985. 19548319 10.1002/cm.20395

[bibr28-1759091414548916] Kontani K. Tada M. Ogawa T. Okai T. Saito K. Araki Y. Katada T (2002) Di-Ras, a distinct subgroup of ras family GTPases with unique biochemical properties. The Journal of Biological Chemistry 277: 41070–41078. 12194967 10.1074/jbc.M202150200

[bibr29-1759091414548916] Kramer E. M. Klein C. Koch T. Boytinck M. Trotter J (1999) Compartmentation of Fyn kinase with glycosylphosphatidylinositol-anchored molecules in oligodendrocytes facilitates kinase activation during myelination. The Journal of Biological Chemistry 274: 29042–29049. 10506155 10.1074/jbc.274.41.29042

[bibr30-1759091414548916] Lee P. R. Cohen J. E. Tendi E. A. Farrer R. De Vries G. H. Becker K. G. Fields R. D (2004) Transcriptional profiling in an MPNST-derived cell line and normal human Schwann cells. Neuron Glia Biology 1: 135–147. 16429615 10.1017/s1740925x04000274 PMC1325299

[bibr31-1759091414548916] Lemke G. Chao M (1988) Axons regulate Schwann cell expression of the major myelin and NGF receptor genes. Development 102: 499–504. 2846259 10.1242/dev.102.3.499

[bibr32-1759091414548916] Li S. Liquari P. McKee K. K. Harrison D. Patel R. Lee S. Yurchenco P. D (2005) Laminin-sulfatide binding initiates basement membrane assembly and enables receptor signaling in Schwann cells and fibroblasts. The Journal of Cell Biology 169: 179–189. 15824137 10.1083/jcb.200501098 PMC2171891

[bibr33-1759091414548916] Li Z. H. Spektor A. Varlamova O. Bresnick A. R (2003) Mts1 regulates the assembly of nonmuscle myosin-IIA. Biochemistry 42: 14258–14266. 14640694 10.1021/bi0354379

[bibr34-1759091414548916] Liffers S. T. Maghnouj A. Munding J. B. Jackstadt R. Herbrand U. Schulenborg T. Hahn S. A (2011) Keratin 23, a novel DPC4/Smad4 target gene which binds 14-3-3epsilon. BMC Cancer 11: 137. 21492476 10.1186/1471-2407-11-137 PMC3095566

[bibr35-1759091414548916] Linder M. E. Deschenes R. J (2007) Palmitoylation: Policing protein stability and traffic. Nature Reviews Molecular Cell Biology 8: 74–84. 10.1038/nrm2084 17183362

[bibr36-1759091414548916] Loebel D. A. Studdert J. B. Power M. Radziewic T. Jones V. Coultas L. Tam P. P (2011) Rhou maintains the epithelial architecture and facilitates differentiation of the foregut endoderm. Development 138: 4511–4522. 21903671 10.1242/dev.063867

[bibr37-1759091414548916] Magnaghi V. Parducz A. Frasca A. Ballabio M. Procacci P. Racagni G. Fumagalli F (2010) GABA synthesis in Schwann cells is induced by the neuroactive steroid allopregnanolone. Journal of Neurochemistry 112: 980–990. 19943853 10.1111/j.1471-4159.2009.06512.x

[bibr38-1759091414548916] Magyar J. P. Ebensperger C. Schaeren-Wiemers N. Suter U (1997) Myelin and lymphocyte protein (MAL/MVP17/VIP17) and plasmolipin are members of an extended gene family. Gene 189: 269–275. 9168137 10.1016/s0378-1119(96)00861-x

[bibr39-1759091414548916] Makoukji J. Shackleford G. Meffre D. Grenier J. Liere P. Lobaccaro J. M. Massaad C (2011) Interplay between LXR and Wnt/beta-catenin signaling in the negative regulation of peripheral myelin genes by oxysterols. The Journal of Neuroscience: The Official Journal of the Society for Neuroscience 31: 9620–9629. 21715627 10.1523/JNEUROSCI.0761-11.2011 PMC6623163

[bibr40-1759091414548916] McKee K. K. Harrison D. Capizzi S. Yurchenco P. D (2007) Role of laminin terminal globular domains in basement membrane assembly. The Journal of Biological Chemistry 282: 21437–21447. 17517882 10.1074/jbc.M702963200

[bibr41-1759091414548916] Moldovan M. Pinchenko V. Dmytriyeva O. Pankratova S. Fugleholm K. Klingelhofer J. Kiryushko D (2013) Peptide mimetic of the S100A4 protein modulates peripheral nerve regeneration and attenuates the progression of neuropathy in myelin protein P0 null mice. Molecular Medicine 19: 43–53. 23508572 10.2119/molmed.2012.00248 PMC3646097

[bibr42-1759091414548916] Monje P. V. Rendon S. Athauda G. Bates M. Wood P. M. Bunge M. B (2009) Non-antagonistic relationship between mitogenic factors and cAMP in adult Schwann cell re-differentiation. Glia 57: 947–961. 19053056 10.1002/glia.20819 PMC2829776

[bibr43-1759091414548916] Napoli I. Noon L. A. Ribeiro S. Kerai A. P. Parrinello S. Rosenberg L. H. Lloyd A. C (2012) A central role for the ERK-signaling pathway in controlling Schwann cell plasticity and peripheral nerve regeneration *in vivo* . Neuron 73: 729–742. 22365547 10.1016/j.neuron.2011.11.031

[bibr44-1759091414548916] Nave K. A. Trapp B. D (2008) Axon-glial signaling and the glial support of axon function. Annual Review of Neuroscience 31: 535–561. 10.1146/annurev.neuro.30.051606.094309 18558866

[bibr45-1759091414548916] Nodari A. Zambroni D. Quattrini A. Court F. A. D'Urso A. Recchia A. Feltri M. L (2007) Beta1 integrin activates Rac1 in Schwann cells to generate radial lamellae during axonal sorting and myelination. The Journal of Cell Biology 177: 1063–1075. 17576799 10.1083/jcb.200610014 PMC2064366

[bibr46-1759091414548916] Ogata T. Iijima S. Hoshikawa S. Miura T. Yamamoto S. Oda H. Tanaka S (2004) Opposing extracellular signal-regulated kinase and Akt pathways control Schwann cell myelination. The Journal of Neuroscience: The Official Journal of the Society for Neuroscience 24: 6724–6732. 15282275 10.1523/JNEUROSCI.5520-03.2004 PMC6729716

[bibr47-1759091414548916] Ory S. Brazier H. Blangy A (2007) Identification of a bipartite focal adhesion localization signal in RhoU/Wrch-1, a Rho family GTPase that regulates cell adhesion and migration. Biology of the Cell/Under the Auspices of the European Cell Biology Organization 99: 701–716. 17620058 10.1042/BC20070058

[bibr48-1759091414548916] Parkinson D. B. Bhaskaran A. Arthur-Farraj P. Noon L. A. Woodhoo A. Lloyd A. C. Jessen K. R (2008) c-Jun is a negative regulator of myelination. The Journal of Cell Biology 181: 625–637. 18490512 10.1083/jcb.200803013 PMC2386103

[bibr49-1759091414548916] Parkinson D. B. Bhaskaran A. Droggiti A. Dickinson S. D'Antonio M. Mirsky R. Jessen K. R (2004) Krox-20 inhibits Jun-NH2-terminal kinase/c-Jun to control Schwann cell proliferation and death. The Journal of Cell Biology 164: 385–394. 14757751 10.1083/jcb.200307132 PMC2172235

[bibr50-1759091414548916] Parkinson D. B. Dickinson S. Bhaskaran A. Kinsella M. T. Brophy P. J. Sherman D. L. Jessen K. R (2003) Regulation of the myelin gene periaxin provides evidence for Krox-20-independent myelin-related signalling in Schwann cells. Molecular and Cellular Neurosciences 23: 13–27. 12799134 10.1016/s1044-7431(03)00024-1

[bibr51-1759091414548916] Peirano R. I. Goerich D. E. Riethmacher D. Wegner M (2000) Protein zero gene expression is regulated by the glial transcription factor Sox10. Molecular and Cellular Biology 20: 3198–3209. 10757804 10.1128/mcb.20.9.3198-3209.2000 PMC85614

[bibr52-1759091414548916] Rigopoulou E. I. Roggenbuck D. Smyk D. S. Liaskos C. Mytilinaiou M. G. Feist E. Bogdanos D. P (2012) Asialoglycoprotein receptor (ASGPR) as target autoantigen in liver autoimmunity: Lost and found. Autoimmunity Reviews 12: 260–269. 22571878 10.1016/j.autrev.2012.04.005

[bibr53-1759091414548916] Saarikangas J. Barral Y (2011) The emerging functions of septins in metazoans. EMBO Reports 12: 1118–1126. 21997296 10.1038/embor.2011.193 PMC3207108

[bibr54-1759091414548916] Sandelin M. Zabihi S. Liu L. Wicher G. Kozlova E. N (2004) Metastasis-associated S100A4 (Mts1) protein is expressed in subpopulations of sensory and autonomic neurons and in Schwann cells of the adult rat. The Journal of Comparative Neurology 473: 233–243. 15101091 10.1002/cne.20115

[bibr55-1759091414548916] Saravanan K. Schaeren-Wiemers N. Klein D. Sandhoff R. Schwarz A. Yaghootfam A. Franken S (2004) Specific downregulation and mistargeting of the lipid raft-associated protein MAL in a glycolipid storage disorder. Neurobiology of Disease 16: 396–406. 15193296 10.1016/j.nbd.2004.03.008

[bibr56-1759091414548916] Schaeren-Wiemers N. Bonnet A. Erb M. Erne B. Bartsch U. Kern F. Suter U (2004) The raft-associated protein MAL is required for maintenance of proper axon–glia interactions in the central nervous system. The Journal of Cell Biology 166: 731–742. 15337780 10.1083/jcb.200406092 PMC2172435

[bibr57-1759091414548916] Schaeren-Wiemers N. Schaefer C. Valenzuela D. M. Yancopoulos G. D. Schwab M. E (1995a) Identification of new oligodendrocyte- and myelin-specific genes by a differential screening approach. Journal of Neurochemistry 65: 10–22. 7790852 10.1046/j.1471-4159.1995.65010010.x

[bibr58-1759091414548916] Schaeren-Wiemers N. Valenzuela D. M. Frank M. Schwab M. E (1995b) Characterization of a rat gene, rMAL, encoding a protein with four hydrophobic domains in central and peripheral myelin. The Journal of Neuroscience: The Official Journal of the Society for Neuroscience 15: 5753–5764. 7643216 10.1523/JNEUROSCI.15-08-05753.1995 PMC6577631

[bibr59-1759091414548916] Schafer D. P. Bansal R. Hedstrom K. L. Pfeiffer S. E. Rasband M. N (2004) Does paranode formation and maintenance require partitioning of neurofascin 155 into lipid rafts? The Journal of Neuroscience: The Official Journal of the Society for Neuroscience 24: 3176–3185. 15056697 10.1523/JNEUROSCI.5427-03.2004 PMC6730037

[bibr61-1759091414548916] Schneider A. Lander H. Schulz G. Wolburg H. Nave K. A. Schulz J. B. Simons M (2005) Palmitoylation is a sorting determinant for transport to the myelin membrane. Journal of Cell Science 118: 2415–2423. 15923654 10.1242/jcs.02365

[bibr62-1759091414548916] Scranton T. W. Iwata M. Carlson S. S (1993) The SV2 protein of synaptic vesicles is a keratan sulfate proteoglycan. Journal of Neurochemistry 61: 29–44. 7685814 10.1111/j.1471-4159.1993.tb03535.x

[bibr63-1759091414548916] Simons K. Toomre D (2000) Lipid rafts and signal transduction. Nature Reviews Molecular Cell Biology 1: 31–39. 10.1038/35036052 11413487

[bibr64-1759091414548916] Sinouris E. A. Skandalis S. S. Kilia V. Theocharis A. D. Theocharis D. A. Ravazoula P. Papageorgakopoulou N (2009) Keratan sulfate-containing proteoglycans in sheep brain with particular reference to phosphacan and synaptic vesicle proteoglycan isoforms. Biomedical Chromatography 23: 455–463. 19101914 10.1002/bmc.1127

[bibr65-1759091414548916] Storbeck C. J. Wagner S. O'Reilly P. McKay M. Parks R. J. Westphal H. Sabourin L. A (2009) The Ldb1 and Ldb2 transcriptional cofactors interact with the Ste20-like kinase SLK and regulate cell migration. Molecular Biology of the Cell 20: 4174–4182. 19675209 10.1091/mbc.E08-07-0707 PMC2754931

[bibr66-1759091414548916] Tao W. Pennica D. Xu L. Kalejta R. F. Levine A. J (2001) Wrch-1, a novel member of the Rho gene family that is regulated by Wnt-1. Genes & Development 15: 1796–1807. 11459829 10.1101/gad.894301 PMC312736

[bibr67-1759091414548916] Tawk M. Makoukji J. Belle M. Fonte C. Trousson A. Hawkins T. Massaad C (2011) Wnt/beta-catenin signaling is an essential and direct driver of myelin gene expression and myelinogenesis. The Journal of Neuroscience: The Official Journal of the Society for Neuroscience 31: 3729–3742. 21389228 10.1523/JNEUROSCI.4270-10.2011 PMC6622795

[bibr68-1759091414548916] Teh M. T. Blaydon D. Ghali L. R. Briggs V. Edmunds S. Pantazi E. Philpott M. P (2007) Role for WNT16B in human epidermal keratinocyte proliferation and differentiation. Journal of Cell Science 120: 330–339. 17200136 10.1242/jcs.03329

[bibr69-1759091414548916] ten Asbroek A. L. Verhamme C. van Groenigen M. Wolterman R. de Kok-Nazaruk M. M. Baas F (2005) Expression profiling of sciatic nerve in a Charcot-Marie-Tooth disease type 1a mouse model. Journal of Neuroscience Research 79: 825–835. 15672449 10.1002/jnr.20406

[bibr70-1759091414548916] Topilko P. Schneider-Maunoury S. Levi G. Baron-Van Evercooren A. Chennoufi A. B. Seitanidou T. Charnay P (1994) Krox-20 controls myelination in the peripheral nervous system. Nature 371: 796–799. 7935840 10.1038/371796a0

[bibr71-1759091414548916] Underwood C. K. Reid K. May L. M. Bartlett P. F. Coulson E. J (2008) Palmitoylation of the C-terminal fragment of p75(NTR) regulates death signaling and is required for subsequent cleavage by gamma-secretase. Molecular and Cellular Neurosciences 37: 346–358. 18055214 10.1016/j.mcn.2007.10.005

[bibr72-1759091414548916] Vicente-Manzanares M. Ma X. Adelstein R. S. Horwitz A. R (2009) Non-muscle myosin II takes centre stage in cell adhesion and migration. Nature Reviews Molecular Cell Biology 10: 778–790. 10.1038/nrm2786 PMC2834236 19851336

[bibr73-1759091414548916] Wang H. Tewari A. Einheber S. Salzer J. L. Melendez-Vasquez C. V (2008) Myosin II has distinct functions in PNS and CNS myelin sheath formation. The Journal of Cell Biology 182: 1171–1184. 18794332 10.1083/jcb.200802091 PMC2542477

[bibr74-1759091414548916] Watanabe Y. Usada N. Minami H. Morita T. Tsugane S. Ishikawa R. Hidaka H (1993) Calvasculin, as a factor affecting the microfilament assemblies in rat fibroblasts transfected by src gene. FEBS Letters 324: 51–55. 8504859 10.1016/0014-5793(93)81530-d

[bibr75-1759091414548916] Xiao J. Kilpatrick T. J. Murray S. S (2009) The role of neurotrophins in the regulation of myelin development. Neuro-Signals 17: 265–276. 19816063 10.1159/000231893

[bibr76-1759091414548916] Zacchetti D. Peranen J. Murata M. Fiedler K. Simons K (1995) VIP17/MAL, a proteolipid in apical transport vesicles. FEBS Letters 377: 465–469. 8549777 10.1016/0014-5793(95)01396-2

[bibr77-1759091414548916] Zhang J. S. Wang L. Huang H. Nelson M. Smith D. I (2001) Keratin 23 (K23), a novel acidic keratin, is highly induced by histone deacetylase inhibitors during differentiation of pancreatic cancer cells. Genes, Chromosomes & Cancer 30: 123–135. 11135429

[bibr78-1759091414548916] Zimmermann H (1996) Accumulation of synaptic vesicle proteins and cytoskeletal specializations at the peripheral node of Ranvier. Microscopy Research and Technique 34: 462–473. 8837022 10.1002/(SICI)1097-0029(19960801)34:5<462::AID-JEMT6>3.0.CO;2-O

[bibr79-1759091414548916] Zoltewicz S. J. Lee S. Chittoor V. G. Freeland S. M. Rangaraju S. Zacharias D. A. Notterpek L (2012) The palmitoylation state of PMP22 modulates epithelial cell morphology and migration. ASN Neuro 4: 409–421. 23127255 10.1042/AN20120045 PMC3563111

[bibr80-1759091414548916] Zvibel I. Wagner A. Pasmanik-Chor M. Varol C. Oron-Karni V. Santo E. M. Kariv R (2013) Transcriptional profiling identifies genes induced by hepatocyte-derived extracellular matrix in metastatic human colorectal cancer cell lines. Clinical & Experimental Metastasis 30: 189–200. 22930170 10.1007/s10585-012-9527-8

